# A Detoxification-Free Process for Enhanced Ethanol Production From Corn Fiber Under Semi-Simultaneous Saccharification and Fermentation

**DOI:** 10.3389/fmicb.2022.861918

**Published:** 2022-03-30

**Authors:** Yingjie Guo, Jiamin Huang, Nuo Xu, Hexue Jia, Xuezhi Li, Jian Zhao, Yinbo Qu

**Affiliations:** State Key Laboratory of Microbial Technology, Shandong University, Qingdao, China

**Keywords:** corn fiber, dilute sulfuric acid pretreatment, optimization, semi-simultaneous saccharification and fermentation, ethanol

## Abstract

Corn fiber, a by-product from the corn-processing industry, is an attractive feedstock for cellulosic ethanol because of its rich carbohydrate content (mainly residual starch, cellulose, and hemicellulose), abundant reserves, easy collection, and almost no transportation cost. However, the complex structure and components of corn fiber, especially hemicellulose, make it difficult to be effectively hydrolyzed into fermentable sugars through enzymatic hydrolysis. This study developed a simple and easy industrialized process without detoxification treatment for high-yield ethanol produced from corn fiber. Corn fiber was pretreated by dilute acid under the conditions optimized by Box-Behnken design (0.5% H_2_SO_4_ at 105°C for 43 min), and 81.8% of total sugars, including glucose, xylose, and arabinose, could be recovered, then the mixture (solid and hydrolysates) was directly used for semi-simultaneous saccharification and fermentation without detoxification, and ethanol yield reached about 81% of the theoretical yield.

## Introduction

Bioethanol, being a kind of fuel made from organic matter resulting from agriculture or forestry, is a sustainable resource with the advantages, such as cleanliness, renewable, and reduced dependence on petroleum imports (Lovett et al., 2011; Toor et al., 2020). In addition, as a fuel octane enhancing additive, bioethanol can diversify energy supplies while contributing significantly to reducing carbon and particle emissions (Moore et al., 2017). Currently, commercial bioethanol is mainly first-generation ethanol produced from starch- or sugar-containing feedstocks, such as corn, other grains, and sugar cane, which require huge cultivatable land while directly competing with the food supply (Nigam and Singh, 2011; Searchinger et al., 2018). The second-generation fuel ethanol uses renewable lignocellulose as raw materials, such as corn stover and other agricultural and forestry residues, but its commercial production has been limited by high production costs, including raw material costs, pretreatment costs, enzyme costs, and investment costs. The cost of raw materials is one of the most important factors affecting the commercial production of second-generation ethanol. Corn fiber is mainly composed of residual starch, cellulose, and hemicellulose. As a residue of the corn processing industry, it is estimated that the annual output of corn fiber reached 10.9 million tons in China (Jiang et al., 2018). In traditional corn ethanol facilities, corn fiber commonly passes through the fermentation and distillation stages and ends up in the distiller’s dried grains with solubles (DDGS) rather than being converted to ethanol, which reduces the total output of corn ethanol (Kurambhatti et al., 2018). Corn fiber is expected to become an attractive feedstock for cellulosic ethanol because of rich carbohydrates, abundant reserves, easy collection, and almost no transportation cost. According to reports, if corn fiber can be economically converted into ethanol, it will increase the total corn ethanol production by up to 13% on the existing basis while improving the protein content of DDGS (Bothast and Schlicher, 2005).

Unlike other lignocellulose, such as corn stover, hemicellulose is the most abundant component of corn fiber (about 40% of dry corn fiber weight), mainly composed of glucuronoarabinoxylan substituted by various side chains (Saha, 2003; Beri et al., 2020). It covers the surface of cellulose, thus affecting the accessibility of cellulase to cellulose. In contrast, because of the complex sugar compositions and structure of hemicellulose in corn fiber and the interaction between different components, it requires a complex hemicellulase system for completing the thorough degradation of hemicellulose (Agger et al., 2010). All the factors make corn fiber difficult to be effectively hydrolyzed into fermentable sugars by direct enzymatic hydrolysis. Therefore, pretreatment before enzymatic hydrolysis has been proposed to improve the enzymatic digestibility of corn fiber for obtaining high fermentable sugar yield (Kumar et al., 2009; Brodeur et al., 2011; Zhang et al., 2021). Till now, some methods used for pretreating corn fiber have been reported. For example, Bura et al. (2002) treated corn fiber by the steam explosion in a batch reactor at various degrees of severity and found that maximum total sugar yields of 81% could be obtained when corn fiber was pretreated at 190°C for 5 min with addition of 6% SO_2_, and using the pretreated corn fiber as substrate, the ethanol concentration reached 6.9 g/L by enzymatic hydrolysis and fermentation at 2% solid content, but the conversion of hemicellulose sugar is lower than 60%. Juneja et al. (2021) performed a two-step pretreatment of corn fiber separated from whole stillage, including liquid hot water pretreatment (LHW) and disk milling. It was shown that, under optimal conditions, the yields of glucose, xylose, and arabinose after enzymatic hydrolysis were 96.18, 72.39, and 66.33%, respectively, but only 21.54 g/L of ethanol concentration was reached after fermentation at 20% solid content. Both steam explosion and LHW require high temperature and pressure, as well as pressure equipment, but the conversion of hemicellulose in pretreated corn fiber was still low. In contrast, the pretreatment conditions of high temperature and high pressure also led to the formation of fermentation inhibitors through further degradation of sugar products.

Many literature showed that dilute acid pretreatment could effectively degrade hemicellulose of lignocellulose materials (Noureddini and Byun, 2010; Jiang et al., 2015; Mikulski and Kłosowski, 2018; Santos et al., 2018), thus the method has been used for pretreating corn fiber to obtain the high conversion of cellulose and hemicellulose, but the monosaccharides would be further degraded during the pretreatment to form inhibitors, such as furfural and 5-hydroxymethylfurfural (HMF). For example, Iram et al. (2019) pretreated DDGS with dilute acid, and the yield of total reducing sugar (TRS) reached 0.382 g/g DDGS; besides TRS, furfural and HMF were also produced during the acid pretreatment. Li et al. (2019) used the combination of acid pretreatment with distillation to *in situ* pretreat whole stillage containing corn fiber and recycled it to the liquefaction step, which improved the ethanol yield by 6.3% compared to the traditional process, and the cellulose conversion was 77.5%, but similarly, many inhibitors were also produced, which led to incomplete fermentation. Noureddini and Byun (2010) used dilute sulfuric acid to treat corn fiber and found that the highest yield of monosaccharides (63.1 g sugar/100 g corn fiber) was obtained when the pretreatment was conducted at 5% of biomass loading (w/w), 1.5% of sulfuric acid concentration (v/v), and 140°C, but the concentration of furfural in pretreatment liquor was also high (3.8 mg/ml) after pretreatment under the conditions. Besides, their results also showed that the formation of furfural could be significantly decreased when pretreatment temperature dropped from 140 to 120°C during the dilute sulfuric acid pretreatment of corn fiber.

Although the inhibitors produced by the dilute acid pretreatment, for example, acetic acid, formic acid, furfural, HMF, and phenolic compounds, limit the applicability of the hydrolysate for subsequent biotransformation (Hendriks and Zeeman, 2009; Yemiş and Mazza, 2011; Lee et al., 2017), and detoxification treatment must be conducted before fermentation to decrease their adverse effects on subsequent saccharification and fermentation, the dilute acid pretreatment is still an attractive approach for improving enzymatic digestibility of corn fiber because of high content and complex structure of hemicellulose in corn fiber. However, the pretreatment process still needs to be further improved to more effectively improve the enzymatic digestibility of corn fiber while minimizing sugar loss and inhibitors formation. To achieve this goal, in this study, we optimized dilute sulfuric acid pretreatment conditions, including temperature, acid concentration, and reaction time, by using the response surface method (RSM), then, the pretreated corn fiber with hydrolysates was directly used for the production of ethanol without detoxification by semi-simultaneous saccharification and fermentation (semi-SSF) process to assess the feasibility of the integrated process for ethanol production using corn fiber as feedstock. In the semi-SSF process, the cellulase from *Penicillium oxalicum*, which has more abundant hemicellulase activities than the cellulase from *Trichoderma reesei* (Gao et al., 2021), was used in the pre-hydrolysis of corn fiber-rich in hemicellulose components and subsequent SSF process, and *Saccharomyces cerevisiae* LF2, an engineered strain with glucose and xylose metabolism, was used in the fermentation of hydrolysates for obtaining high ethanol yield by simultaneous conversion of glucose and xylose to ethanol.

## Materials and Methods

### Materials and Strains

Corn fiber was obtained from Juneng Golden Corn Co. Ltd., (Shouguang, China). It was ground using an MF 10 basic Microfine grinder (IKA, Germany) with a 2.0-mm-hole diameter sieve, then stored in a sealed polyethylene bag at −20°C before use.

The liquid chromatography standards, including glucose, galactose, arabinose, xylose, mannose, galacturonic acid, glucuronic acid, furfural, HMF, and acetic acid, were purchased from Sigma-Aldrich Co. LLC (St. Louis, MO, United States). Reagent grade sulfuric acid (98%), anhydrous sodium acetate, sodium hydroxide, and ethanol were obtained from Sinopharm Chemical Reagent Co. Ltd. (Shanghai, China).

*Penicillium oxalicum* MCAX, an engineered strain in our laboratory (Gao et al., 2021), was used to produce cellulases.

*Saccharomyces cerevisiae* LF2 is capable of metabolizing xylose and glucose was maintained in our group. It was provided by Professor Xiaoming Bao from Qilu University of Technology, Shandong.

### Analysis of Chemical Compositions of Corn Fiber

The chemical compositions of corn fiber were determined according to the National Renewable Energy Laboratory (NREL) protocols. The extractives were quantified according to the analytical method NREL/TP-510-42619 (Sluiter et al., 2005a), and the ash was determined by the incineration method described by Sluiter et al. (2005b). The contents of cellulose, hemicellulose (xylose, arabinose, and galactose), lignin, and acetyl group were determined by two-step hydrolyzing corn fiber with H_2_SO_4_ of 72% (w/w) and 4% (w/w), respectively, according to the analytical method NREL/TP-510-42618 (Sluiter et al., 2008a). The starch was quantified according to the analytical method NREL/TP-510-42619 (Sluiter and Sluiter, 2005). The protein was quantified according to the analytical method NREL/TP-510-42625 (Hames et al., 2008). The sample collected during the determination of acetyl group content was filtered by 0.22 μm Millipore Syringe Filters (Jinteng, China), and the filtrate was used to analyze glucuronic acid by high-performance liquid chromatography (HPLC) (Shimadzu, Japan), which was equipped with Shimadzu’s 20A refractive index detector. An Aminex HPX-87H column (300 × 7.8 mm) (Bio-Rad, Richmond, CA, United States) was used to separate the compounds at 60°C. The mobile phase was 0.005 M H_2_SO_4_ with a 0.5 ml/min flow rate.

### Enzyme Preparation

The cellulase used in this study was produced by *P. oxalicum* MCAX according to the method suggested by Gao et al. (2021). The strain MCAX was cultivated in 50 ml of seed medium [g/L, wheat bran: 20, peptone: 10, glucose: 10, (NH_4_)_2_SO_4_: 2, KH_2_PO_4_: 3, and MgSO_4_: 0.5] in 300 ml Erlenmeyer flasks for 24 h at 30°C and 200 rpm on a rotary shaker. Then, the culture was inoculated into a fermentation medium [g/L, wheat bran: 30, bean cake powder: 15, microcrystalline cellulose: 30, (NH_4_)_2_SO_4_: 2, KH_2_PO_4_: 5, and MgSO_4_: 0.5] with an inoculation ratio of 10% (v/v) and incubated at 30°C and 200 rpm for 6 days. After fermentation, the supernatant was obtained by centrifugation at 10,000 rpm for 10 min and used as crude cellulase. The hydrolytic activities of the crude cellulase were as follows: filter paper activity (FPA) 6 stocktickerFPU/ml, xylanase 783 IU/ml, arabinofuranosidase 5 IU/ml, and β-xylosidase 1 IU/ml.

### Pretreatment of Corn Fiber

Pretreatment of corn fiber with dilute sulfuric acid was performed in a laboratory-scale vertical pressure steam sterilizer (Deqiang, China). A total of 4 g of corn fiber and dilute acid solution was thoroughly mixed in 50 ml flasks with 12 ml of reaction volume. Based on preliminary experiments, the following experimental conditions were tested: temperature of 105–125°C, H_2_SO_4_ concentration of 0.2–0.5% (w/v), and time of 10–60 min. The experiments were designed by a Box-Behnken RSM using Design-Expert version 8.0.6.1 software and are shown in Table 2.

For the significance analysis of different factors in the pretreatment, *p*-value was obtained by ANOVA and used for determining statistically significant. *p* > 0.05 indicates that the factor is not significant; *p* ≤ 0.05 indicates that it is significant; and *p* ≤ 0.01 indicates that it is very significant.

After pretreatment, the flasks were cooled down to room temperature. A part of the pretreated corn fiber slurries was collected to analyze the contents of furfural, HMF, formic acid, and acetic acid.

### Saccharification

The saccharification was performed at 5% (w/v) substrate concentration in 50 ml flasks with a reaction volume of 20 ml. The pretreated corn fiber slurries were first adjusted to pH 5.0 with 10 M NaOH solution, mixed well with crude cellulase solution and acetate buffer of pH 4.8 in flasks, and incubated at 48°C in a thermostat air bath shaker set at 150 rpm. The samples were taken at reaction times of 0, 12, 24, and 48 h, respectively, and 72 h to analyze the concentrations of monomeric sugars in the enzymatic hydrolysates.

All experiments were performed in triplicate, and the average values are given in this study.

### Acclimation of Yeast for Obtaining Resistant Strain

The hydrolysate of corn fiber was treated with dilute acid under the optimum pretreatment conditions, which contains 4.60 g/L glucose, 3.92 g/L xylose, 0.44 g/L furfural, 1.04 g/L formic acid, and 1.62 g/L acetic acid. For acclimation of the yeast, four different acclimation media were used. One of the media consisted of hydrolysate that was not diluted. The three other media were prepared by diluting hydrolysate to a concentration of 25, 50, and 75% (v/v), respectively. In each acclimation medium, 15.0 g/L glucose, 0.40 g/L (NH_4_)_2_SO_4_, and 1.00 g/L yeast extract were added. *S. cerevisiae* LF2 was first inoculated into YPD medium for activation of 24 h at 30°C, then take 2 ml of inoculum, centrifuge, and transfer the yeast cells to a 50 ml Erlenmeyer flask containing 20 ml of the acclimation medium with the lowest inhibitors concentration and incubated at 30°C to the glucose content in the medium less than 90% of the initial glucose content. After that, the strain was further transferred to the next acclimation medium with a higher inhibitors’ concentration and cultured according to the procedure above until the highest inhibitors concentration. To make the yeast better adapt to the change of inhibitor concentration, the acclimation of yeast in each inhibitor concentration was repeated more than four times.

### Semi-Simultaneous Saccharification and Fermentation

The inoculum was prepared by growing *S. cerevisiae* LF2 in a YPD medium containing 20 g/L glucose, 20 g/L peptone, and 10 g/L yeast extract at 30°C, 200 rpm for 24 h in a thermostat air bath shaker. The inoculum with an OD of 2.0 at 600 nm was used as seed culture, and the inoculation volume was 5% (v/v).

The semi-SSF experiment was performed in a 50 ml sealed Erlenmeyer flask by a rubber plug with a needle. First, the pretreated corn fiber slurries were adjusted to pH 5.0 with 10 M NaOH, and the pre-hydrolysis was conducted at 20% (w/v) solid consistency, 48°C, pH 5.0 for 12 h using cellulase of 10 FPU/g (DM). After pre-hydrolysis, the reaction system temperature was adjusted to 30°C, and 5% (v/v) of seed was inoculated to the reaction system. Fermentation was carried out in a thermostat air bath shaker with a rotation rate of 200 rpm. The samples were taken periodically to determine the concentrations of glucose, xylose, and ethanol.

### Analytical Methods

The FPA and the activities of xylanase, β-xylosidase (*p*NPXase), and α-arabinofuranosidase (*p*NPAase) of culture supernatants were measured using a Whatman No.1 filter paper, insoluble xylan (beech), *p*-nitrophenyl-β-D-xylopyranoside, and *p*-nitrophenyl-α-L-arabinofuranoside as the substrates, respectively, according to the methods presented by Gao et al. (2021). One unit (U) of enzyme activity was defined as the amount of enzyme that liberates 1 μmol of reducing sugars (for FPA and xylanase) equivalent or *p*-nitrophenol (for *p*NPAase and *p*NPXase) per minute under the assay conditions.

The samples collected in the pretreatment, enzymatic hydrolysis, and fermentation stages, respectively, were centrifuged at 10,000 rpm for 10 min. The supernatants were used for analyzing the contents of glucose, xylose, formic Acid, acetic acid, furfural, HMF, and ethanol using HPLC (LC-20AT, refractive index detector RID-20A, Shimadzu, Kyoto, Japan) With an Aminex HPX-87H column (Bio-Rad, Hercules, CA, United States) at 60°C using a mobile phase of 5 mM H_2_SO_4_ at a rate of 0.5 ml/min (Sluiter et al., 2008b). For the samples from the enzymatic hydrolysis and fermentation stage, the supernatants were first boiled for 10 min for inactivating enzyme. The sugar yields (%) were calculated according to the following formula:


(1) $$\%{\text{Yield}}_{\text{Sugar}}=\left({\text{C}}_{\text{HPLC}}\times \text{V}\times \text{CF}\right)/(\text{W}\times {\text{Content}}_{\text{Sugar}}\times 1000)\times 100,$$

where *C*_HPLC_ is the concentration of sugar as determined by HPLC, mg/ml; *V* is the volume of the reaction system, ml; CF (conversion factor) is 0.9 for C-6 sugars and 0.88 for C-5 sugars; *W* is the weight of the substrate in the reaction system, g; and Content_Sugar_ is the percentage of the polymeric sugar in corn fiber.

## Results and Discussion

### Chemical Compositions of Corn Fiber

Table 1 provides the chemical compositions of corn fiber. The corn fiber contains starch, cellulose, and hemicellulose with a total carbohydrate content of 71.22 ± 1.27%. The major components were hemicellulosic sugars (xylose, arabinose, and galactose), and their total contents reached about 40%. These data were consistent with the previously reported results (Kim et al., 2008; Eylen et al., 2011). Compared with the chemical components of corn stover and corn cob (Eylen et al., 2011), the corn fiber had similar total glucan content to corn stover (30.6%) and corn cob (34.8%) and similar xylan content to corn stover (19%). But unlike corn cob and corn stover, a considerable part of the glucose in corn fiber was derived from starch, not just cellulose. In contrast, the contents of other hemicellulose sugars in corn fiber were significantly higher than that in corn stover and corn cob, which means that the degradation of corn fiber may require a more complex enzyme system with more abundant hemicellulase components.

**TABLE 1:** Composition of corn fiber (%, dry basis).

Components	Contents
Starch	13.50 ± 1.24
Cellulose	18.54 ± 1.71
Xylan	20.73 ± 1.79
Arabinose	14.49 ± 1.15
Galactose	3.96 ± 0.32
Lignin	4.07 ± 0.45
Extractives	4.43 ± 0.23
Ash	0.55 ± 0.14
Protein	8.25 ± 0.06
Acetyl group	2.28 ± 0.12
Uronic acid	0.93 ± 0.12

**TABLE 2:** Pretreatment factors and experiment results in the experiments designed by the central composite design from response surface methodology.

Run	Factors			Responses (mg/g)*					
		
	Temp. (°C)	Time (min)	Acid conc. (%, w/v)	Glucose	Xylose	Arabinose	Formic acid	Acetic acid	Furfural
1	125	10	0.35	328	132	110	5.27	9.13	3.93
2	115	60	0.50	333	151	111	5.58	13.6	2.70
3	115	10	0.50	344	125	109	5.37	7.87	2.19
4	115	10	0.20	329	89.5	92.0	5.06	5.07	2.66
5	105	35	0.20	318	91.5	92.4	4.83	3.84	2.35
6	125	35	0.50	340	171	117	5.97	14.5	3.11
7	115	35	0.35	345	135	113	5.39	8.25	2.93
8	105	60	0.35	354	122	111	5.01	5.85	2.78
9	115	60	0.20	328	119	105	5.08	6.43	3.10
10	105	35	0.50	354	131	114	5.02	7.58	2.26
11	115	35	0.35	339	136	112	5.48	9.71	3.13
12	125	35	0.20	329	126	108	5.12	7.08	3.23
13	115	35	0.35	347	133	112	5.42	7.97	3.20
14	115	35	0.35	338	130	109	5.41	7.70	3.07
15	125	60	0.35	334	162	114	6.05	13.8	3.73
16	115	35	0.35	326	127	107	5.14	7.74	3.02
17	105	10	0.35	310	89.5	89.8	4.88	4.09	2.03

*Based on per gram dry corn fiber.

### Pretreatment of Corn Fiber With Dilute Sulfuric Acid

**FIGURE 1:**
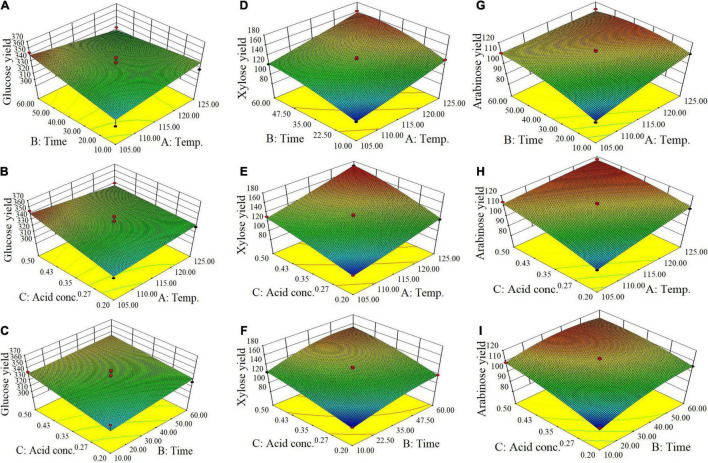
Response surface plots of yields of glucose **(A–C)**, xylose **(D–F)**, and arabinose **(G–I)** (mg/g corn fiber) obtained from the Box-Behnken design of RSM.

From batch kinetics studies, it was found that the factors affecting acid pretreatment included the type of biomass, the type of acid, acid concentration, reaction time, and reaction temperature (Rahman et al., 2006). Among them, the operating conditions, including reaction time, temperature, and acid concentration, would significantly affect the efficiency of dilute acid pretreatment (López-Arenas et al., 2010). In this study, RSM incorporating variables of temperature, time, and acid concentration was used to optimize the pretreatment process parameters for maximizing the enzymatic conversion of carbohydrate in corn fiber to monosaccharides and minimizing the formation of inhibitors. The complete list of runs and responses is provided in Table 2. Five experimental runs were replicated at the center point of the design to confirm any differences in the estimation procedure as a measure of precision. Table 2 shows the yields of formic acid, acetic acid, and furfural after pretreatment and the yields of glucose, xylose, and arabinose after pretreatment and following enzymatic hydrolysis. Figure 1 shows the response surface plots of glucose, xylose, and arabinose yields under different pretreatment conditions. ANOVA of the models for glucose, xylose, arabinose, furfural, formic acid, and acetic acid is given in Table 3. The regression equations of different products were as follows, respectively.

**TABLE 3:** ANOVA Table of the adjusted models from dilute sulfuric acid pretreated and enzyme hydrolyzed corn fiber.

Source	Sum of squares	DF	Mean square	*F* value	*p*-Value
*Glucose*					
Model	1301.93	6	216.99	2.19	0.1304
Residual	989.62	10	98.96		
Lack of fit	711.74	6	118.62	1.71	0.3146
*R* ^2^	0.5681				
*Xylose*					
Model	1.156E-003	9	1.284E-004	85.43	<0.0001
Residual	1.052E-005	7	1.504E-006		
Lack of fit	4.006E-006	3	1.335E-006	0.82	0.5470
*R* ^2^	0.9910				
*Arabinose*					
Model	1023.70	9	113.74	14.80	0.0009
Residual	53.78	7	7.68		
Lack of fit	23.89	3	7.96	1.07	0.4572
*R* ^2^	0.9501				
*Furfural*					
Model	3.94	9	0.44	11.19	0.0022
Residual	0.27	7	0.039		
Lack of fit	0.23	3	0.077	7.24	0.0429
*R* ^2^	0.9350				
*Formic* acid					
Model	1.70	6	0.28	15.66	0.0001
Residual	0.18	10	0.018		
Lack of fit	0.11	6	0.019	1.07	0.4951
*R* ^2^	0.9038				
*Acetic* acid					
Model	156.64	6	26.11	65.27	<0.0001
Residual	4.00	10	0.40		
Lack of fit	1.23	6	0.20	0.30	0.9105
*R* ^2^	0.9751				

Glucose = + 334.95 – 0.49 × Temp. + 4.69 × Time + 8.45 × Acid conc. – 9.53 × Temp. × Time – 6.38 × Temp. × Acid conc. – 2.60 × Time × Acid conc.

Xylose = + 132.21 + 19.61 × Temp. + 14.86 × Time + 19.06 × Acid conc. – 0.64 × Temp. × Time + 1.19 × Temp. × Acid conc. – 0.66 × Time × Acid conc. + 1.56 × Temp.2 – 7.19 × Time 2 – 3.91 × Acid conc.2

Arabinose = + 110.82 + 5.20 × Temp. + 4.97 × Time + 6.71 × Acid conc. – 4.23 × Temp. × Time −3.00 × Temp. × Acid conc. – 2.97 × Time × Acid conc. – 0.62 × Temp.2 – 4.26 × Time 2 – 2.29 × Acid conc.2

Furfural = + 3.07 + 0.57 × Temp. + 0.19 × Time – 0.14 × Acid conc. – 0.24 × Temp. × Time – 7.500E-003 × Temp. × Acid conc. + 0.018 × Time × Acid conc. + 0.061 × Temp.2 – 0.014 × Time 2 – 0.39 × Acid conc.2

Formic acid = + 5.30 + 0.33 × Temp. + 0.14 × Time + 0.23 × Acid conc. + 0.16 × Temp. × Time + 0.17 × Temp. × Acid conc. + 0.047 × Time × Acid conc.

Acetic acid = + 8.25 + 2.90 × Temp. + 1.69 × Time + 2.65 × Acid conc. + 0.73 × Temp. × Time + 0.93 × Temp. × Acid conc. + 1.10 × Time × Acid conc.

where sugars and inhibitors appear as yield (mg/g) and the variables are Temp. (temperature, °C), Time (in min), and Acid conc. (acid concentration, %, w/v).

It is shown in Figure 1 and *p*-values in ANOVA that the yield of glucose obtained from the enzymatic hydrolysis of pretreated corn fiber was sensitive to acid concentration (*p* = 0.0372 ≤ 0.05) but not to pretreatment time (*p* = 0.2119 > 0.05) and temperature (*p* = 0.8922 > 0.05), but the yield of xylose was significantly very sensitive to reaction time (*p* < 0.0001), temperature (*p* < 0.0001), and acid concentration (*p* < 0.0001). Initially, the increase in pretreatment temperature is in favor of the conversion of xylan to xylose in subsequent enzymatic hydrolysis, but a high pretreatment temperature led to the dehydration reaction of xylose accelerated, thus decreased xylose production but increased furfural content (Table 2), which is similar to the results reported in some literature (Kabel et al., 2007; Noureddini and Byun, 2010).

In contrast, it was found that the yields of glucose and xylose (based on the original content in corn fiber) were 10.5 and 38.3%, respectively, in the pretreatment stage, and the yields of glucose and xylose were 95.5 and 72.4% after enzymatic hydrolysis, indicating that xylan was more dissolved out in the pretreatment stage. Avci et al. (2013) also reported the difference in production behavior between glucose and xylose. They found that after being treated with dilute sulfuric acid a considerable part of xylan was directly hydrolyzed into xylose, while glucose was mostly produced by enzymatic hydrolysis. This is similar to our results. In the study, maximum xylose of 171 mg/g corn fiber was obtained by pretreatment at 125°C for 35 min using 0.5% (m/v) H_2_SO_4_. Similar to xylose, as shown in Figure 1I, the yield of arabinose was also sensitive to pretreatment temperature (*p* = 0.0011 ≤ 0.01), time (*p* = 0.0014), and acid concentration (*p* = 0.0002).

Overall, for three main sugars in corn fiber (glucose, xylose, and arabinose), the highest yield of total sugars was produced by pretreatment at 125°C for 35 min using 0.5% (w/v) H_2_SO_4_ and enzyme hydrolysis, including glucose of 340 mg, xylose of 171 mg, and arabinose of 117 mg, respectively, on the basis of per gram corn fiber. This amount was approximately 85.4% of the three sugars present in the corn fiber.

Furfural and HMF were generated from xylose and glucose in the pretreatment process through a further dehydration reaction, respectively (Jönsson and Martín, 2016). It was found that furfural formation was also most sensitive to pretreatment temperature (*p* < 0.0001), but not to acid concentration (*p* = 0.949 > 0.05), maybe because sulfuric acid only acts as a catalyst to provide an acidic environment. The highest furfural content of 3.93 mg/g corn fiber was found after pretreatment at 125°C for 10 min with 0.35% sulfuric acid (Table 2). Extending pretreatment time from 10 min to 60 min under conditions of 0.35% sulfuric acid and 125°C, furfural content in pretreatment liquid decreased, maybe due to further decomposition of furfural. It was also found that the yield of formic acid, a degradation product of furfural (Yang et al., 2012), increased (Table 2). In addition, Yemiş and Mazza (2011) also reported that the too-long reaction time could cause the condensation reaction of furfural to form other products. However, no HMF was detected in any hydrolysate from the acid pretreatment process, possibly because little glucose was degraded during the acid pretreatment under pretreatment conditions used in the paper. Acetic acid was also detected in hydrolysates after the acidic pretreatment (Table 2), which comes from the shedding of acetyl groups in hemicellulose (Ibbett et al., 2011), and its content was also sensitive to reaction time (*p* < 0.0001), temperature (*p* < 0.0001), and acid concentration (*p* < 0.0001). Longer reaction time, higher reaction temperature, and acid concentration can promote acetic acid formation.

It was found that, although the pretreatment can obtain the highest amounts of sugars at 125°C for 35 min using 0.5% H_2_SO_4_, there was also the higher production of formic acid (5.97 mg/g corn fiber), acetic acid (14.5 mg/g corn fiber), and furfural (3.11 mg/g corn fiber), which may result in the inhibitory effect on microbial growth in subsequent ethanol fermentation. To obtain the maximum sugars while minimizing the formation of inhibitory compounds as much as possible, based on the RSM analysis (Figure 1 and Tables 2, 3), it was determined that the suitable pretreatment conditions for the corn fiber were the acid concentration of 0.5%, reaction temperature of 105°C, and reaction time of 43 min. Under this condition, the yield of glucose, xylose, and arabinose reached 354 mg/g corn fiber, 133 mg/g corn fiber, and 114 mg/g corn fiber, respectively, which is equivalent to 81.8% of the total amount of the three sugars present in the corn fiber. Although the yield (81.8%) was slightly lower than the highest sugar yield (85.4%) obtained at 125°C for 35 min using 0.5% H_2_SO_4_, the amounts of inhibitors produced in the mild pretreatment significantly decreased, for example, decreased by 25.7% for furfural, 45.6% for acetic acid, and 14.2% for formic acid, respectively.

### Semi-Simultaneous Saccharification and Fermentation of Pretreated Corn Fiber for Producing Ethanol

#### Assessment of Inhibition Effect of Hydrolysate

**FIGURE 2:**
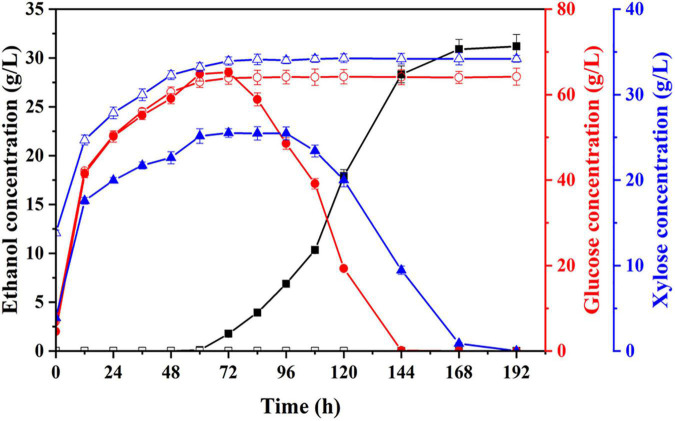
Changes in concentrations of ethanol, glucose, and xylose during fermentation of pretreated corn fiber by dilute acid under different pretreatment conditions for assessing inhibitors’ effect on fermentation. Ethanol, square; glucose, circle; xylose, triangle; pretreatment with 0.5% H_2_SO_4_ at 105°C for 43 min, solid; and pretreatment with 0.5% H_2_SO_4_ at 125°C for 35 min, hollow.

According to the reports from the literature, furfural concentration greater than 0.2 mg/ml or acetic acid concentration greater than 5 mg/ml could be severely toxic to the growth of *S. cerevisiae* (Sanchez and Bautista, 1988; Larsson et al., 1999; Kim et al., 2013). In the system of multiple inhibitors coexisting, the interaction of these inhibitors could enhance the toxicity, leading to the greater inhibition of microbial growth and fermentation than any single inhibitor (Palmqvist and Hahn-Hägerdal, 2000; Hou et al., 2018). To high efficiently produce ethanol using the pretreated corn fiber as substrate, in this study, using two hydrolysates from corn fiber pretreated by dilute acid under optimum conditions (0.5% H_2_SO_4_, 105°C for 43 min) and the conditions for obtaining the highest total sugars yields (0.5% H_2_SO_4_, 125°C for 35 min), respectively, we first assessed the inhibition effect of hydrolysates on the growth of *S. cerevisiae* LF2 by investigating the effect on ethanol fermentation. The hydrolysates were preadjusted to pH 5.0 with 10 M NaOH solution, and *S. cerevisiae* LF2 was inoculated into the hydrolysates. It was shown in Figure 2 that the growth of *S. cerevisiae* LF2 was obviously inhibited in the hydrolysates, in which the growth of *S. cerevisiae* LF2 was strongly inhibited in the hydrolysate from pretreated corn fiber at 125°C using 0.5% (w/v) H_2_SO_4_ for 35 min, and almost no ethanol was produced in the whole fermentation period. For the hydrolysate from corn fiber pretreated in optimum conditions, ethanol was just produced after about 60 h of fermentation.

#### Acclimation and Fermentation Assessment of Yeast

**FIGURE 3:**
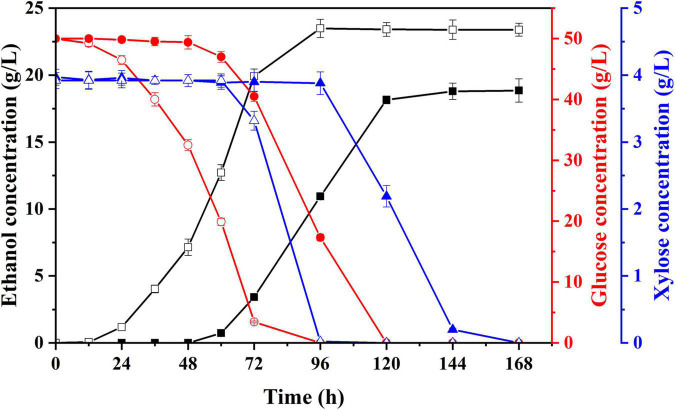
Changes in concentrations of ethanol, glucose, and xylose of parent strain (solid) and domesticated yeast (hollow) in acclimation medium with the highest inhibitors concentration supplemented with glucose. Ethanol, square; glucose, circle; xylose, triangle.

To shorten the lag growth period of *S. cerevisiae* LF2 during semi-SSF of hydrolysate, strain domestication was conducted by continuously and gradually increasing hydrolysate concentration in acclimation medium for making the *S. cerevisiae* LF2 adapt to this environment with inhibitors. After multiple habituated cultures, the tolerance of *S. cerevisiae* LF2 to inhibitors was greatly improved. The performances of the domesticated strain and parent strain were compared by cultivation in the acclimation media with the highest concentration of inhibitors, in which glucose was supplemented to 50.0 g/L in the media. As shown in Figure 3, using the domesticated strain, the ethanol concentration in fermentation liquid was significantly increased compared to the parent strain (23.5 g/L *vs.* 18.8 g/L), and the lag period decreased from 60 to 12 h, which could meet the requirements of semi-SSF, thus, in the following experiments, the domesticated strain was directly applied to ethanol fermentation of pretreated corn fiber without pre-detoxification treatment.

#### Semi-Simultaneous Saccharification and Fermentation of Pretreated Corn Fiber for Ethanol Production

**FIGURE 4:**
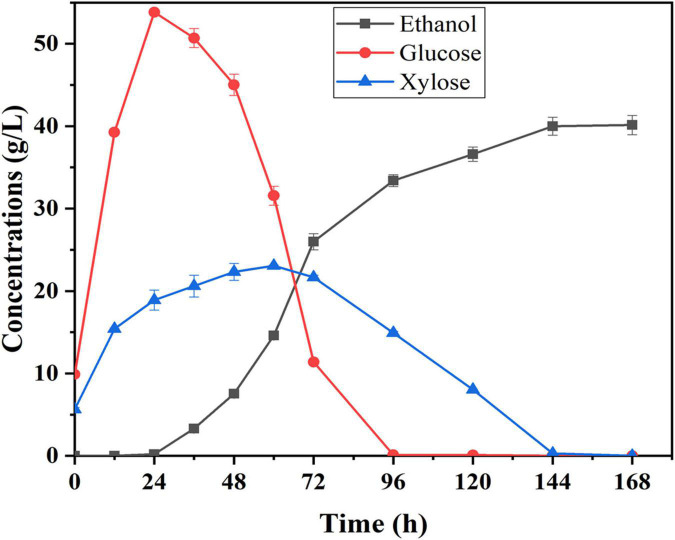
Time courses of sugar utilization and ethanol production during semi-SSF with *S. cerevisiae* LF2 and corn fiber pretreated at 105°C; 0.5% H_2_SO_4_ for 43 min; fermentation conditions: 20% (w/v) of solid content, 30°C and pH 5.0.

Semi-simultaneous saccharification and fermentation was used to evaluate ethanol production potential using corn fiber as substrate under the best pretreatment conditions. Compared to the SSF process, a pre-hydrolysis step was first carried out prior to SSF under the optimal conditions of enzymatic hydrolysis, which help to promote enzymatic hydrolysis and liquefaction of cellulosic substrate. The pretreated corn fiber was pre-hydrolyzed with the cellulase preparation for 12 h at 48°C and pH 5.0 before yeast was inoculated. Figure 4 shows the changes in glucose, xylose, and ethanol concentrations concerning time during the semi-SSF. It was shown that traces of ethanol in fermentation liquid (0.20 g/L) began to be detected at 24th hour, and after that, glucose was continuously consumed up to the concentration of 0 at 96th hour. Xylose concentration started decreasing when its concentration was close to that of glucose, and it was consumed completely at the end of fermentation. Finally, the ethanol concentration of 40.14 g/L was obtained, and ethanol yield was approximately 81% of the theoretical yield. However, in this study, the total fermentation time was long (about 144 h), and the rate of xylose conversion was still low. Thus, in further work, the *S. cerevisiae* LF2 will be further improved to decrease the lag period of strain, and the enzyme system will be modified to improve the rate of xylose conversion.

Some results from literature about cellulosic ethanol production using corn fiber as feedstock through different pretreatment and saccharification-fermentation process are summarized in Table 4. It was shown that, in the existing research, detoxification treatments, such as water washing, biodetoxification, and overliming, were usually used after acidic and alkaline pretreatment. For example, Shrestha et al. (2010) used water washing to detoxify the mild NaOH or steam pretreated corn fiber, and the ethanol concentration of 3.30 and 2.66 g/L, respectively, was obtained after fermentation under the condition of 4.3% solid content. However, its solid content and final ethanol concentration were too low for practical application. Gáspár et al. (2007) also used water washing for detoxification of corn fiber pretreated by KOH. The ethanol concentration reached 12.50 g/L after fermentation at 5% solid content by semi-SSF process. However, because most of the hemicellulose was dissolved in the alkali pretreatment process and wasted after water washing, the ethanol yield was very low, only 0.062 g/g initial corn fiber. Bura et al. (2002) reported that the inhibitors in the hydrolysate could be completed removed by detoxifying steam explosion-pretreated corn fiber with overliming treatment, but fermentation was conducted at only 2% of solid content, which was too low to be suitable for practical application. Although water washing and overliming can remove inhibitors, they also increase process steps and costs while generating problems, such as wastewater and the loss of sugars. Different from this, Zhang et al. (2021) adopted biodetoxification by inoculating *Paecilomyces variotii* to remove inhibitors produced by citric acid pretreatment, and the ethanol concentration reached 70.2 g/L using semi-SSF at 25% of solid content, and ethanol production reached 0.280 g/g initial corn fiber. Although less wastewater is produced due to biological detoxification rather than water washing and overliming, high citric acid dosage (4%) and reaction temperature (165°C) during pretreatment and too-long biodetoxification as strain growth and inactivation after detoxification influenced the economy of the process. Li et al. (2019) used the combination of acid pretreatment with distillation to *in situ* pretreat whole stillage containing corn fiber. The pretreated stillage was recycled to the liquefaction step with the addition of cellulase, or hydrolyzed using cellulase and then recycled to the liquefaction step, or hydrolyzed using cellulase followed by C6/C5 sugar co-fermentation. Compared with the traditional dry milling process, the highest ethanol yield increased by 6.3%, and the conversion of cellulose reached 77.5%. However, a large number of inhibitors in the pretreatment liquid resulted in incomplete fermentation. Myat and Ryu (2014) used thermal-mechanical extrusion to pretreat corn fiber, which reduced the crystallinity and polymerization degree of cellulose in corn fiber, and the final ethanol concentration reached 29.08 g/L. Juneja et al. (2021) used the combination of liquid hot water pretreatment with wet disk milling to pretreat corn fiber, resulting in a final ethanol concentration of 21.54 g/L at 20% of solid content. Due to the lack of chemical pretreatment, there were fewer inhibitors in these two pretreatments so that ethanol fermentation could be carried out without detoxification. But these pretreatments required higher energy consumption because of mechanical milling and high temperature compared to the process used in this study, and the final ethanol concentration was also lower than that in this study.

**TABLE 4:** Comparison of cellulosic ethanol production from corn fiber feedstock.

Pretreatment condition	Detoxification	Fermentation process	Solid content (w/v) (%)	Dosage of enzymes (per gram dry matter)	Ethanol production (g/g)	Ethanol concentration (g/L)	References
Alkaline pretreatment with 2% (w/w) NaOH at 30°C, for 2 h; Steaming at 100 °C for 2 h, 20% (w/w)	Water washing	SSF	4.3	10 FPU Spezyme CP	0.077*; 0.062*	3.30; 2.66	Shrestha et al., 2010
Alkaline pretreatment with 1% KOH at 120°C for 1 h under pressure (2 bar)	Water washing	Semi-SSF	5	25 FPU Celluclast 1.5 L and 25 IU Novozyme 188	0.062**	12.50	Gáspár et al., 2007
Steam explosion with 6% SO_2_ at 190°C for 5 min	Overliming	SHF	2	8.3 FPU Celluclast 1.5 L, 10.1 IU Novozym 188 and 50-80 IU glucoamylase	0.490*	6.89	Bura et al., 2002
Acid pretreatment with 4% citric acid at 165°C for 2 min	Biodetoxification	Semi-SSF	25	10 FPU Cellic CTec2	0.280**	70.20	Zhang et al., 2021
Extrusion (300 rpm, 140°C)	None	Semi-SSF	7.1	5.9 FPU Cellulclast 1.5 L, 38.0 CBU β-glucosidase, 0.8 FBG Viscozyme L	0.410*	29.08	Myat and Ryu, 2014
Liquid hot water pretreatment at 180°C for 10 min and wet disk milling	None	SSF	20	0.05 g Cellulase	0.11*	21.54	Juneja et al., 2021
Liquid hot water at 160°C for 20 min	Water washing	SHF	7.8	5 FPU Novozyme 188 and 5 FPU Celluclast 1.5L	0.260*	20.00	Mosier et al., 2005
Acid pretreatment with 0.5% H_2_SO_4_ at 105°C for 43 min	None	Semi-SSF	20	10 FPU MCAX	0.201**	40.14	This study

*g/g pretreated corn fiber; **g/g initial corn fiber, calculated from the mass balance data provided in these literature.

Overall, this study proposed a simple and low-cost process used to produce ethanol from corn fiber. In this process, mild pretreatment with dilute acid can enable the saccharification and fermentation process to directly be conducted without detoxification while ensuring the low loss of sugars during pretreatment, which was conducive to ensure the high ethanol yield, high final ethanol concentration (as high solid content after pretreatment), and no wastewater problem in pretreatment stage. These advantages show that this process has a good prospect of industrial application.

## Conclusion

This study developed a simple production process for cellulosic ethanol using corn fiber. In this process, an optimized mild dilute acid pretreatment process ensured the high sugar recovery and fewer inhibitor production while high enzymatic digestibility of substrate, and the mixtures of solid and hydrolysates from pretreatment were directly transported into subsequent enzymatic hydrolysis and fermentation process without detoxification treatment for achieving high-efficiency conversion of sugar to ethanol and obtaining high ethanol yield at 20% of solid content through the semi-SSF process using acclimated yeast. This process could be easily industrialized with low equipment investment, fewer process steps, and no pretreatment wastewater problem.

## Data Availability Statement

The raw data supporting the conclusions of this article will be made available by the authors, without undue reservation.

## Author Contributions

YG and JZ designed the study. YG performed the experiment, analyzed the data, and wrote the manuscript. JH, NX, and HJ participated in part of the experiments. YQ revised the manuscript. JZ and XL conceived the study and edits for the manuscript. All authors contributed to the creation of the manuscript.

## Conflict of Interest

The authors declare that the research was conducted in the absence of any commercial or financial relationships that could be construed as a potential conflict of interest.

## Publisher’s Note

All claims expressed in this article are solely those of the authors and do not necessarily represent those of their affiliated organizations, or those of the publisher, the editors and the reviewers. Any product that may be evaluated in this article, or claim that may be made by its manufacturer, is not guaranteed or endorsed by the publisher.
